# Assessing Presenting Symptoms, Co-Morbidities, and Risk Factors for Mortality in Underserved Patients With Non-Hereditary Early-Onset Colorectal Cancer

**DOI:** 10.7759/cureus.16117

**Published:** 2021-07-02

**Authors:** Shravani Reddy, Awf Mouchli, Lindsey Bierle, Miranda Gerrard, Chirstopher Walsh, Adil Mir, David P Lebel, Christopher Mason, Douglas Grider, Marrieth Rubio

**Affiliations:** 1 Internal Medicine, Carilion Clinic, Roanoke, USA; 2 Gastroenterology, Cleveland Clinic, Cleveland, USA; 3 Medical Student, Internal Medicine, Virginia Tech Carilion School of Medicine, Roanoke, USA; 4 Pathology, Virginia Tech Carilion School of Medicine, Roanoke, USA; 5 Pathology, Carilion Roanoke Memorial Hospital, Roanoke, USA; 6 Basic Science Education, Virginia Tech Carilion School of Medicine, Roanoke, USA; 7 Gastroenterology and Hepatology, Virginia Tech Carilion School of Medicine, Roanoke, USA

**Keywords:** early-onset colorectal cancer, clinical symptoms, co-morbid conditions, rectal bleeding, risk factors

## Abstract

Background

The presenting symptoms and co-morbidities contributing to mortality in young patients (age < 50 years old) with colorectal cancer (CRC) are poorly understood. We reviewed these features in our patient population with non-hereditary early-onset CRC (EO-CRC).

Study aim

This study aimed to assess characteristics of patients with a diagnosis of non-hereditary EO-CRC, including presenting symptoms and metabolic disorders contributing to mortality in underserved areas of southwest Virginia.

Methods

In this retrospective observational study, we selected patients aged 18-50 years with a diagnosis of non-hereditary EO-CRC from 2008 to 2016 at Carilion Roanoke Memorial Hospital. The electronic medical record was queried to identify demographic data, medical history, histopathology results, lab values, and mortality. The cumulative risks of symptoms and co-morbid metabolic disorders was estimated using Kaplan-Meier curves.

Results

We identified 139 patients with non-hereditary EO-CRC (mean age 41.6 ± 6.9 years). Almost half of these patients were obese (BMI > 30), 30.9% had a diagnosis of hypertension, 29% had hyperlipidemia (HLD), and 17.35% had diabetes mellitus type 2 (DM2). Diagnosis was delayed by 4.5 months from initial presentation, and 17% had advanced disease (stage III/IV). Also, 68.5% of patients were symptomatic with one to three symptoms, most commonly with rectal bleeding (45.3%). The chronicity of HLD (≥5 years) was associated with reduced survival in our patients with EO-CRC. The survival of females with multiple metabolic disorders was reduced compared to females with a single metabolic disorder.

Conclusions

Multiple symptoms, chronic HLD, and female gender with multiple metabolic disorders were factors associated with poor outcomes in non-hereditary EO-CRC patients.

## Introduction

Colorectal cancer (CRC) is the third most common cancer and the second most common cause of cancer-related mortality in the United States (U.S.) [1]. Since the mid-1990s, the incidence and mortality of CRC in older populations (> 50 years old) within the U.S. have decreased [1,2]. However, the incidence of CRC has increased by 2% per year in young patients (<50 years old) [2-4]. CRC in young patients is often associated with late diagnosis, unfavorable pathology, and poor outcomes [3]. The disease characteristics and risk factors associated with the mortality of early-onset CRC (EO-CRC) have not been elucidated.

Current screening guidelines identify those at risk of CRC based on age, family history, or a predisposing condition (inflammatory bowel disease or hereditary syndromes). However, more than half of patients with EO-CRC do not have a known family history, hereditary condition, or inflammatory bowel disease [3,5-7].

In the absence of a family history or predisposing hereditary syndrome, current screening guidelines do not account for patients at risk of CRC under the age of 45 years. The U.S. Preventive Services Task Force (USPSTF) has recently updated screening CRC screening guidelines to include individuals aged 45 years and older [8-10]. In individuals younger than 45 years old, identification of suspicious symptoms may be the only way to facilitate an earlier diagnosis of CRC. There is a need to identify the presentation of symptoms, which would impact survival, facilitate early diagnosis, and likely have implications on screening guidelines and treatment recommendations.

An understanding of co-morbid conditions can also help identify risk of EO-CRC diagnosis and mortality. The rise in non-hereditary EO-CRC is thought to be associated with several factors such as obesity, western diet, alcohol and smoking use, physical inactivity, and environmental factors [7,11-13]. With an increasing incidence of obesity, there is a parallel increase in metabolic disorders such as diabetes mellitus type 2 (DM2), hyperlipidemia (HLD), and hypertension (HTN). Metabolic disease is associated with chronic low-grade inflammation, compromised immunity, oxidative stress, and alterations of gut microbiota, all of which are also considered risk factors for CRC [14,15].

Within the U.S., the incidence of EO-CRC is highest in southern and southeastern regions [2,3]. Our hospital, Carilion Roanoke Memorial Hospital (CRMH), is in Central Appalachia, an area characterized by poverty, obesity, unemployment, and poor access to health care. Here, there is a higher incidence and mortality of gastrointestinal cancers compared to other comparable regions within the U.S. [3,16-19].

We have observed a rise in young patients with non-hereditary EO-CRC in our patient population at CRMH in southwest Virginia. Clinical observations from CRMH have identified the increasing mortality of young patients with CRC and prompted us to suspect that there are underappreciated factors contributing to mortality in this patient population. Better understanding of factors associated with non-hereditary EO-CRC diagnosis and mortality may help clarify targets for improvement in screening and diagnosis.

## Materials and methods

Study population

We performed a single-center retrospective observational study of patients with a diagnosis of EO-CRC at CRMH, a tertiary care referral center, from August 1, 2008, till December 31, 2016, with follow-up through the end of 2018. We included all patients aged 18 to 49 years with a histopathologic diagnosis of CRC and collected data regarding demographics, medical history, drug, tobacco and alcohol use, lab values, and histopathology including microsatellite instability (MSI), *KRAS* and *BRAF* mutations, and mismatch repair (MMR) expression (*MSH2*, *MLH1*, *MSH6*, *PMS2*) from the electronic medical record (EMR). Additional chart review was used to gather information regarding metabolic disorders, duration of disease, and disease control. Patients with diagnoses of inflammatory bowel disease, family history of colon cancer, and known hereditary conditions such as Lynch syndrome, familial adenomatous polyposis, and MYH-associated polyposis were excluded.

Defining symptom onset

Symptom onset was measured from initial evaluation. A query of symptoms and their frequency was documented and extracted from the EMR. We queried symptoms from initial gastroenterology consultation, including abdominal pain, diarrhea, constipation, weight loss, rectal bleeding, rectal pain, bloating, nausea and vomiting, and others (including fatigue, fever, pelvic pain, syncope, flu-like symptoms). Anemia was also included as a sign of microscopic blood loss. It was measured from hemoglobin after initial consultation defined as hemoglobin of <13 g/dL in men and <12 g/dL in women.

Defining metabolic disease

In our chart review, we used cutoffs for each metabolic disorder, duration of disease, and disease control. A diagnosis with DM2 was defined as an initial A1c > 6.5% and control defined as A1c < 8% within the follow-up period. HTN was defined as at least two ambulatory blood pressure (BP) readings with values greater than 129/89, with control defined as BP <140/90 with at least one antihypertensive agent in subsequent visits. HLD was defined as an low-density lipoprotein (LDL) > 160 mg/dL and control defined as LDL < 100 mg/dL. Disease duration was defined as having the disease for 0-5 years or >5 years. In addition, body mass index (BMI) was calculated and categorized as underweight (BMI < 18.5), normal (BMI 18.5-24.9), overweight (BMI >25-29.9), obese (BMI 30-39.9), and morbidly obese (BMI >40).

Statistical analysis

The data were reported as mean (± SD), median (interquartile range [IQR]), ranges, and categorical variables by counts and percentages as appropriate. Estimates of the mortality rates in patients with several presenting symptoms and patients with metabolic diseases were determined using the Kaplan-Meier survival curve with a log-rank test.

## Results

Patient eligibility and demographics

A total of 280 patients aged 18-49 years were identified with a diagnosis of CRC. After the exclusion criteria, as noted in the Methods section, were applied, 139 patients with non-hereditary EO-CRC were identified. The mean age at the time of diagnosis was 41.6 ± 6.9 years, with 53.2% males. Almost half of the patients were obese (30.2%) or morbidly obese (16.3%). Additional patient characteristics describing alcohol and substance use are given in Table 1.

**Table 1:** Patient Characteristics

Characteristics			Missing Data
Age	Mean age 41.6 ± 6.9 years		0
Gender	Male	74/139 (53.2%)	
	Female	65/139 (46.8%)	
BMI	Underweight (BMI <18.5)	6/129 (4.65%)	10
	Normal (BMI 18.5-24.9)	21/129 (16.3%)	
	Overweight (BMI 25-29)	42/129 (32.6%)	
	Obese (BMI 30-39.9)	39/129 (30.2%)	
	Morbidly obese (BMI >40)	21/129 (16.3%)	
Alcohol use	Active	49/122 (40.2%)	17
	History of use	12/122 (9.8%)	
Tobacco use	Active	23/122 (18.9%)	17
	History of use	20/122 (16.4%)	
Illicit drug use	Active	3/121 (2.5%)	18
	History of use	3/121 (2.5%)	

Frequency and long-term survival associated with symptoms

On initial evaluation, 20.1% of patients were asymptomatic, 68.5% of patients reported one to three signs or symptoms, and 11.5% of patients had three or more signs or symptoms. Rectal bleeding was the most common presenting symptom in 45.3% of patients followed by abdominal pain (36%) and diarrhea (23%) of patients. Additional symptoms and their frequency are reported in Tables 2, 3.

**Table 2:** Frequency of Signs and Symptoms

Signs and Symptoms	Frequency
Rectal bleeding	63/139 (45.3%)
Abdominal pain	50/139 (36%)
Diarrhea	32/139 (23%)
Constipation	26/139 (18.7%)
Anemia	24/139 (17.3%)
Weight loss	24/139 (17.3%)
Other symptoms	18/139 (12.9%)
Nausea with vomiting	15/139 (10.8%)
Rectal pain	3/139 (2.2%)
Bloating	3/139 (2.2%)

**Table 3:** Frequency of Symptoms at the Time of Diagnosis

Symptoms at the Time of Diagnosis	Frequency
Asymptomatic	28/139 (20.1%)
1-3 symptoms	95/139 (68.5%)
≥3 symptoms	16/139 (11.5%)

**Figure 1:**
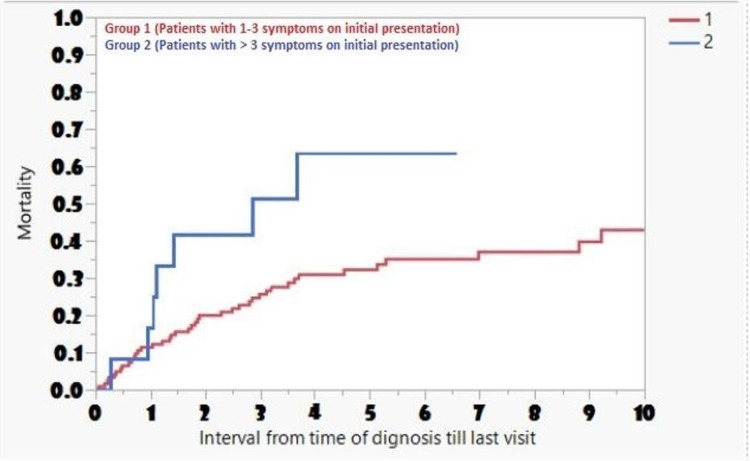
Frequency and Long-Term Survival Associated with Symptoms

A diagnosis of CRC was established after a mean of 4.5 ± 11.4 months, with roughly 17% presenting with advanced stage disease (III/IV). Median time of survival was lower in patients with more signs and symptoms (more than three) on initial presentation compared to those with one to three symptoms (2.8 vs. 13.7 months; p = 0.036) (Figure 1).

Frequency and long-term survival associated with metabolic disorders

At the time of diagnosis, 48% were obese or morbidly obese (BMI > 30). Forty-three (30.9%) patients had HTN, 88.6% were well controlled, and 6.8% had the disease for more than five years. Forty (29.0%) patients had HLD, 34.1% were controlled, and 41.3% had the disease for more than five years. DM2 was diagnosed in 17.3% of patients, 72% were controlled, and 48% had the disease for more than five years. Incidence and chronicity of metabolic disease are detailed in Table 4.

**Table 4:** Frequency and Duration of Metabolic Disorders

	Disease Present	Controlled	0-5 years	6-10 years
Hypertension	43/139 (30.9%)	37/43 (86.0%)	40/43 (93%)	3/43 (6.9%)
Hyperlipidemia	40/139 (28.8%)	14/40 (35%)	24/40 (60%)	13/40 (32.5%)
Diabetes mellitus type 2	24/139 (17.3%)	17/24 (70.8%)	14/24 (58.3%)	10/24 (41.7%)

**Figure 2:**
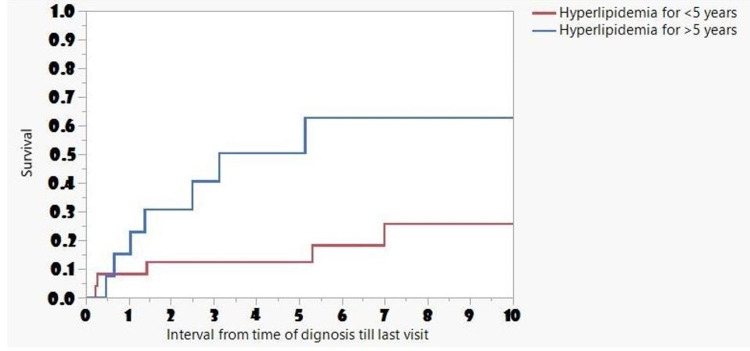
Frequency and Long-Term Survival Associated with Metabolic Disorders

The median time of survival decreased in patients with chronic HLD (more than five years) compared to patients with HLD (less than five years) (3.11 vs. 13.7; p=0.04) (Figure 2).

**Figure 3:**
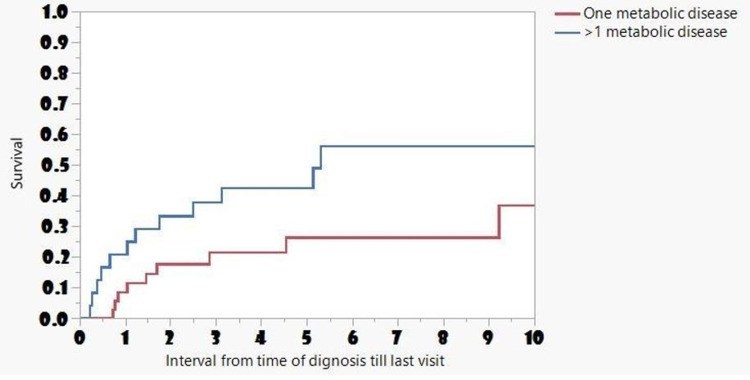
Frequency and Long-Term Survival Associated with Metabolic Disorders in Females

Females with more than one metabolic disease had worse survival compared to females with a single metabolic disease (p=0.04) (Figure 3).

## Discussion

In this study evaluating underserved patients with non-hereditary EO-CRC, we found that the diagnosis of EO-CRC was delayed by 4.5 months and 17% of the patients had advanced disease (stage III/IV). The presence of multiple presenting symptoms, chronic HLD, and females with multiple metabolic disorders were risk factors associated with poor survival in patients with non-hereditary EO-CRC. Our results could help clarify risk factors associated with poor outcomes and identify targets for improved screening and earlier diagnosis.

Roughly 50% of EO-CRC is sporadic and is not associated with a family history of CRC or predisposing condition such as inflammatory bowel disease or hereditary syndromes [3,5-7]. Although factors such as birth weight, early-life body fat percentage, and physical inactivity have been shown to be associated with increased risk of EO-CRC and traditional CRC (T-CRC; age > 50 years), there are limited data regarding whether the metabolic disorders are associated with poor survival in this patient population [5,19,20]. In addition, identifying quantitative targets of metabolic disorders would clarify risk factors associated with mortality in EO-CRC.

A review of prior studies has demonstrated conflicting evidence regarding the association of metabolic disorders and CRC mortality. For example, several studies have demonstrated an association between obesity and DM2 and both T-CRC and EO-CRC mortality [2,21]. Similar to our population, Yeo et al. demonstrated that obesity and DM2 were not risk factors contributing to EO-CRC mortality compared to patients with T-CRC [12]. Although we concluded that chronic HLD was associated poor mortality in EO-CRC, the relationship between cholesterol and CRC mortality had previously been controversial. Kim et al. described an increased rate of advanced colorectal neoplasm in young patients with HLD [22]. We did not observe an associated increase in EO-CRC mortality in patients with a diagnosis of HTN, which is supported by prior studies evaluating the association of HTN with T-CRC mortality that have also shown no relationship [23-25]. However, other studies support that there is an inverse relationship between cholesterol and CRC mortality [26-28]. These discrepancies illustrate that the etiology of EO-CRC is likely multifactorial and further studies are required to identify major contributing risk factors.

Our study also highlights an increasingly common finding that females with multiple metabolic disorders have poorer survival compared to those with a single metabolic disorder. This supports a meta-analysis performed in 2011, which stated that the association of metabolic disorders and EO-CRC was highest in female patients [14]. In addition, Liu et al. conducted a prospective study of women aged 25-42 years from 14 U.S. states and discovered that obesity and weight gain were associated with increased risk of EO-CRC [20]. The women identified at risk were also more symptomatic and had more advanced tumors on presentation. In comparison, a single-center study performed in a U.S. veteran population with average age of 66 years found that men with multiple co-morbidities of metabolic and cardiovascular disorders had an earlier age of diagnosis of CRC, which prompts further assessment in early-onset CRC populations in general [23].

The knowledge of heralding symptoms associated with poor survival in EO-CRC would help prompt investigation of colorectal cancer at an earlier stage. In our patient population, diagnosis of non-hereditary EO-CRC was delayed by an average of 4.5 months compared to prior studies with longer time to diagnosis [29,30]. We confirmed that rectal bleeding is often the most common symptom preceding diagnosis in patients with EO-CRC [29,31]. This parallels a large study by Dozois et al. which found that rectal bleeding was the most common finding in patients with EO-CRC and associated with advanced stage disease and poor mortality [31]. Our results support the conclusion that diagnostic colonoscopy should be offered earlier for patients with more signs or symptoms, especially those with rectal bleeding as this could impact overall survival and facilitate earlier diagnosis.

Our study highlights the importance of considering factors such as symptoms and co-morbidities in tools calculating CRC risk. Outside of screening recommendations, there are few validated tools available to help calculate CRC risk. The National Cancer Institute (NCI) developed a screening calculator, which takes into account age, race, diet, physical activity, and social risk factors such as alcohol and tobacco use [32]. Wells et al. performed a multi-ethnic cohort study analyzing the use of the NCI CRC risk calculator and found adequate accuracy in an average risk population [33]. However, the NCI CRC risk calculator does not account for presenting symptoms or the presence of metabolic disorders such as DM2, HTN, and HLD. Further understanding of symptoms associated with poor survival and the impact of chronic metabolic disease on EO-CRC in prospective studies can be used in conjunction with other risk factors to facilitate earlier diagnosis and predict survival in this population.

The retrospective nature of this study and the small sample size are certainly limitations, as well as the fact that we were not able to collect data on patients who were admitted previously to other facilities in order to assess medication compliance and those who followed up elsewhere. We were also not able to collect data on some variables such as birth weight, body fat percentage, physical activity, or diets due to logistical reasons. High birth weight has been associated with an increased risk of CRC [34]. In addition, demographic information on race was also limited within our EMR, and several studies and guidelines have illustrated the increased incidence of CRC in African American populations [8-10]. Lastly, we relied on vitals collected primarily at outpatient visits and could not account for BP while at home. As white coat HTN is a well-documented phenomenon, this may also serve as a limitation when assessing hypertensive disease as a co-morbidity [35]. These factors could affect the impact of metabolic disease on survival. It should be noted that this study was conducted prior to the updated colon cancer screening guidelines set forth by the USPSTF in 2021.

## Conclusions

In conclusion, various risk factors were found to be associated with poor EO-CRC survival in our underserved patient population including female gender with more than one metabolic factor and symptoms at presentation including rectal bleeding. Future prospective studies are needed to further define quantitative targets for metabolic disease as well as assess other risk factors such as environmental or occupational exposures. Future studies addressing these factors are imperative given the increasing prevalence of obesity and metabolic disease worldwide and the increasing prevalence of metabolic disorder and CRC. Hopefully, further understanding of the quantitative targets of disease can be used to assess risk in patients with EO-CRC and improve primary preventative strategies as well as development of risk stratification tools.
